# Supplementary material for: The effects of genital myiasis on the diversity of the vaginal microbiota in female Bactrian camels
Source: BMC Vet Res. 2022 Mar 5;18:87. doi: 10.1186/s12917-022-03189-5 (PMC8897907; doi:10.1186/s12917-022-03189-5)
Supplement: Supplementary file 5 — Additional file 5. [file 12917_2022_3189_MOESM5_ESM.zip › MPL201709200_16s_yy/Treat1/css/js_css/jsPDF-master.zip!/jsPDF-master/examples/annotation/test_annotation.pdf]

Table of Contents

Page 2
Page 3
Page 4
Page 5
Page 6
Page 7
Page 8
Page 9

Goto First Page
Goto External URL

Page 3

Goto First Page
Goto External URL

Goto First Page
Goto External URL

Goto First Page
Goto External URL

Goto First Page
Goto External URL

Goto First Page
Goto External URL

Goto First Page
Goto External URL

Goto First Page
Goto External URL
